# A systematic review of surgical and interventional radiology procedures for pediatric idiopathic intracranial hypertension

**DOI:** 10.3389/fped.2024.1466688

**Published:** 2024-10-30

**Authors:** Sofia Friso, Vittoria Giacobbo, Luca Mattia Toscano, Beatrice Baldo, Chiara Guariento, Fabrizio Lacarra, Jacopo Norberto Pin, Claudio Ancona, Stefano Sartori, Francesco Causin, Irene Toldo

**Affiliations:** ^1^ Pediatric Neurology and Neurophysiology Unit, Department of Women’s and Children’s Health, University Hospital of Padua, Padua, Italy; ^2^ Division of Neuropediatric, Institute of Pediatrics of Southern Switzerland, Bellinzona, Switzerland; ^3^ Neuroradiology Unit, Department of Women’s and Children’s Health, University Hospital of Padua, Padua, Italy

**Keywords:** idiopathic intracranial hypertension, CSF shunting, ONSF, VSS, review, children and adolescents

## Abstract

**Background:**

Idiopathic intracranial hypertension (IIH) is defined as elevated intracranial pressure and consequent symptoms (mainly headache and visual deterioration) occurring in the absence of secondary causes. Surgical and interventional radiology procedures should be considered for refractory IIH and mainly include cerebrospinal fluid (CSF) diversion techniques, optic nerve sheath fenestration (ONSF), and venous sinus stenting (VSS). Our study aims to review the current literature on the application of these techniques in clinical practice.

**Methods:**

A systematic literature review on the surgical and interventional radiology treatment of IIH was conducted, focusing on ONSF, VSS, and CSF diversion techniques. According to PRISMA guidelines, all reports published in PubMed in the last 30 years (1993–2023) were considered, and among 722 papers, 48 were included in the present study, resulting in a total study population of 454 children or adolescents (11 months–17 years old).

**Results:**

Among 454 patients, 193 underwent an invasive approach, divided into CSF diversion (115/193), ONSF (65/193), VSS (11/193), cranial subtemporal decompression (8/193), and internal cranial expansion (9/193). Sixteen of the 193 patients (8%) required reintervention due to relapsing symptoms or surgical complications, particularly those who underwent CSF diversion. Furthermore, 9/115 required shunt revision due to shunt obstruction or malfunction. We extracted data on the outcome of each procedure: of the 193 patients, 71 experienced a positive outcome with symptom resolution or improvement, while 27 demonstrated a negative outcome.

**Discussion and conclusions:**

Severe and refractory cases of IIH are eligible for invasive treatments. CSF diversion is the most frequently used technique, despite its high failure risk and need for reintervention. ONSF has shown good results in terms of outcome and safety, particularly in children with visual symptoms. VSS is the most recent approach, indicated in children with stenosis of the venous sinus. In our study population, VSS demonstrated good results in terms of symptom resolution and need for reintervention, but its use remains limited to a few centers.

**Systematic Review Registration:**

https://www.crd.york.ac.uk/, PROSPERO (CRD42024504244).

## 1 Introduction

Idiopathic intracranial hypertension (IIH) is characterized by signs and symptoms of elevated intracranial pressure (ICP), without any pathological findings on neuroimaging (except for signs of high ICP) and with a normal composition of the cerebrospinal fluid (CSF) (1). The incidence of IIH in pediatric age ranges between 0.60 and 0.71 per 100,000 children (2, 3). The most frequent symptoms in children are visual loss and headache, often associated with diplopia, tinnitus, and VI cranial nerve palsy (4, 5).

First described by Dandy in 1937 in a work of 22 patients, the diagnostic criteria included signs and symptoms consistent with elevated intracranial pressure and an opening pressure >25 cm H_2_O, normal cerebrospinal fluid composition, and normal to small ventricles without evidence of an intracranial mass on pneumoencephalography (6). These criteria were updated in 1985 by Smith to reflect advancements in neuroimaging (7). The current diagnostic criteria include papilledema, normal neuroimaging, elevated intracranial pressure (≥28 cm H_2_O if the child is under sedation in the lateral decubitus position or ≥25 cm H_2_O if the child is non-sedated or obese), and normal cerebrospinal fluid constituents (8, 9).

Currently, the first-line treatment of pediatric IIH is acetazolamide, aimed at reducing elevated intracranial pressure (10). Most children respond to treatment with acetazolamide [around 91% in the adult population, whereas data are not available for children (11)]. However, some of them show refractory intracranial hypertension or relapsing IIH after discontinuing treatment. Some of these cases require second-line medical treatment or surgical intervention, especially when there is a rapid worsening in visual acuity (12).

This study aimed to review the pertinent literature about the most frequently used invasive treatments of IIH in children: venous sinus stenting, optic nerve sheath fenestration, lumboperitoneal and ventriculoperitoneal shunting, and cranial decompression. We analyzed each technique describing its prevalence in the study population, the need for reintervention, and the outcome in terms of resolution, persistence, or worsening of symptoms.

## 2 Materials and methods

We performed a systematic literature review on the invasive treatment of pediatric IIH focusing on VSS, ONSF, and CSF shunting techniques (i.e., ventriculoperitoneal shunt and lumboperitoneal shunt).

The research was conducted in the PubMed database, complying with PRISMA guidelines (13).

The search terms used were as follows: ((pseudotumor cerebri) OR (idiopathic intracranial hypertension)) AND ((treatment) OR (management)) AND (children). Language restrictions were applied: papers written in English, French, Spanish, or German were included. The available articles were manually filtered, considering patients of pediatric age (<18 years old) who had been diagnosed with IIH [according to the original Dandy's criteria in 1937 (6, 8)] and who underwent an invasive approach (optic nerve sheath fenestration, venous sinus stenting, CSF shunting, cranial decompression), from 1993 to November 2023. This time frame was selected because we believe it is representative of current approaches to IIH and it balances modern and consolidated expertise on the topic. Demographic, clinical, diagnostic, and treatment data were collected.

**Figure 1:**
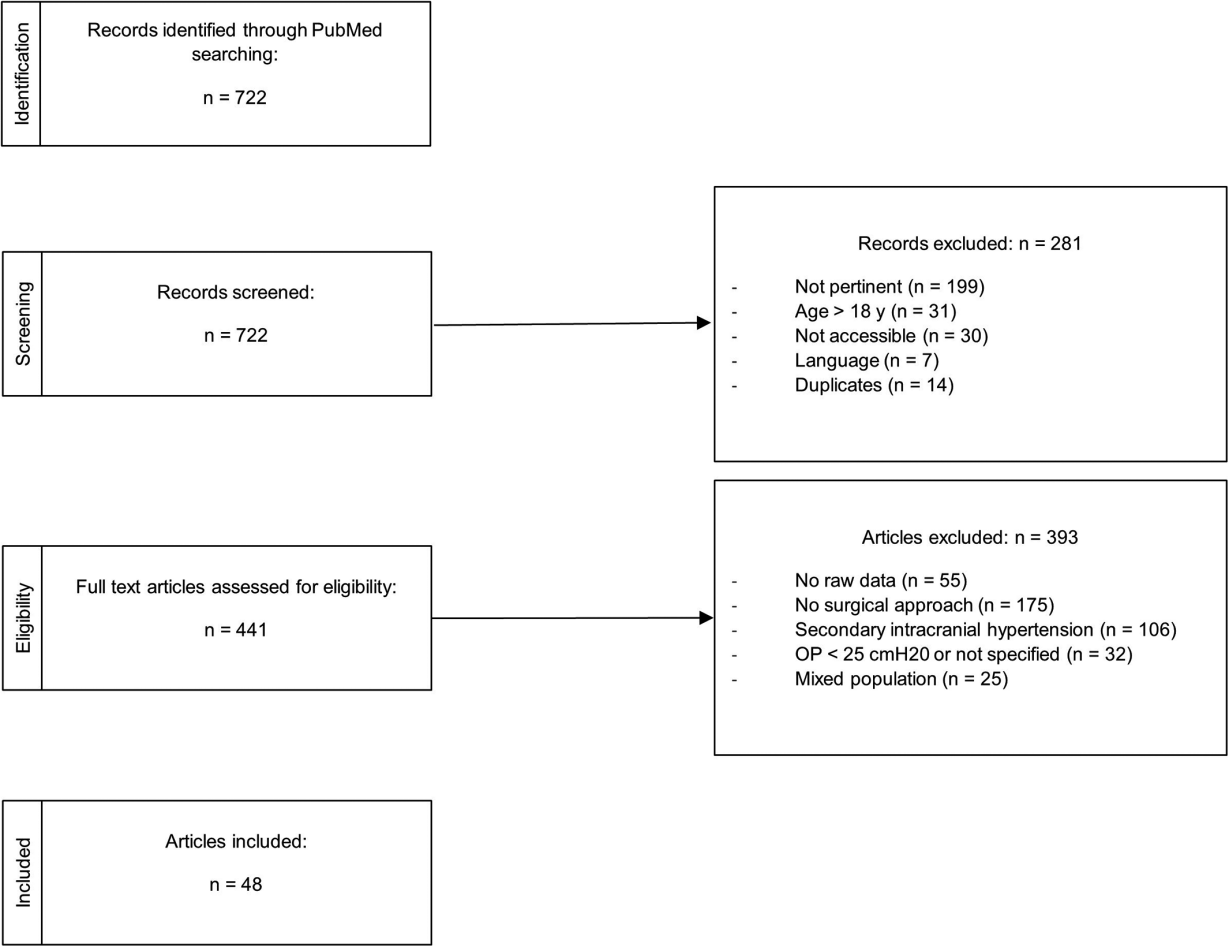
Methods and materials of paper selection.

Studies with mixed populations (where raw data were not clearly defined) or without CSF measurement were excluded. Narrative reviews (those that did not report raw data) were also excluded (Figure 1). A total of 722 records were identified through literature research. Of these, 37 articles were excluded (impossible to download, written in languages other than Italian, English, German, French, and Spanish), and 14 were duplicates (i.e., questions to the author). Among the remaining 671 records, we excluded 230 papers because they either referred to the adult population (31 articles) or were not pertinent to the topic (199 studies). A total of 441 full-text articles were assessed for eligibility. After full-text examination, 393 more records were further excluded for various reasons: 55 did not report raw data; 175 were not relevant to the aim of our review (i.e., did not refer to neurosurgical treatment); 32 studies did not meet the definition of IIH according to Dandy's criteria (1937), particularly if opening pressure was not measured or was inferior than 25 cm H_2_O. A total of 106 records were excluded because they referred to secondary intracranial hypertension, and 25 records were excluded because they did not differentiate the results by age, making it impossible to extract data specific to pediatric patients. A final number of 48 studies were included in the current systematic review, corresponding to a study population of 454 children or adolescents. An *ad hoc* dataset containing the data collected was created (see Supplementary Materials). Only 193 out of 454 children underwent an invasive treatment; the result is that the study population comprises both patients receiving medical treatment and those eligible for an invasive approach. We aimed to broaden the study population to better reflect the proportions found in the general population, despite potential selection bias. We considered the different lines of intervention, the need for reintervention, and the final outcome in terms of resolution, worsening, or persistence of symptoms. In some studies, it was not possible to extract complete information and data regarding the different aspects analyzed in the review (e.g., fundus oculi examination, neuroimaging, medical therapy, and outcome). In these cases, the incomplete data were counted and reported in the results section as missing data. Our systematic review was registered in the PROSPERO system with protocol number CRD42024504244.

## 3 Results

### 3.1 Epidemiology, clinical presentation, and neuroimaging

Considering the records included, a total of 454 pediatric patients were recruited for our study population. The mean age of the study population was 12 years (11 months—17 years); 305 (66%) patients were female.

The most common onset symptoms were headache (352/454, 77.5%), diplopia (104/454, 23%), visual acuity reduction (63/454, 14%), VI nerve palsy (42/454, 9%), tinnitus (74/454, 16%), nausea and vomiting (60/454, 13%), and neck or shoulder pain (78/454, 17%). The clinical features at onset have been summarized in Table 1.

**Table 1:** Clinical manifestation at onset in our study population.

Symptoms at onset	Prevalence (*N*/454)
Headache	352 (77.5%)
Visual symptoms	
• Diplopia	104 (23%)
• Reduction of visual acuity	63 (14%)
• Blurry vision	112 (25%)
• Reduction of visual field	40 (9%)
• Transient visual obscuration	69 (15%)
• Strabismus	6 (1%)
Muscular symptoms	
• Neck or shoulder pain	78 (17%)
• Stiff neck	5 (1%)
Tinnitus	74 (16%)
Nausea and/or vomiting	60 (13%)
VI nerve palsy	42 (9%)
Dizziness and/or vertigo	15 (3%)
Impairment of consciousness	4 (1%)

Unilateral or bilateral papilledema was detected in 377 of 417 patients who underwent fundus oculi examination (90%), while it was negative in 40 of these patients. The ophthalmologic exam was not mentioned in the remaining 37 patients of the total population.

We included only pediatric cases with direct measurement of intracranial hypertension and an opening pressure of >25 cm H_2_O. The mean opening pressure from lumbar puncture was 37.5 cm H_2_O based on data calculated on 393 subjects. In the remaining 61/454 patients, data were aggregated and only provided range values, even though they respected Dandy's criteria (OP > 25 cm H_2_O).

Neuroimaging was performed in 421/454 subjects (93%): among them, 8/421 (2%) subjects underwent only brain computed tomography (CT), 145/421 (34%) only brain magnetic resonance imaging (MRI), and 72/421 patients (17%) both brain CT and brain MRI. In the remaining 196/421 (46%) patients, details about the type of neuroimaging were not available.

A total of 217/421 patients underwent MRI showing indirect hypertension radiological signs only in 59/421 of them (16%). MR cerebral venography (MRV) was performed in 117 patients (54%) revealing abnormalities (primarily venous sinus narrowing or stenosis) in 11% of patients. MRV was not mentioned in the remaining patients (100/217, 46%).

In 362/421 (86%) patients, neuroimaging did not report any significant pathological findings. Among the remaining patients, the most common neuroradiological signs were narrowing/stenosis of the cerebral venous sinuses (15/59, 25%), empty sella sign (13/59, 22%), and optic nerve tortuosity–enlargement (16/59, 27%). More than one radiological sign was present in 6/59 (10%). A comprehensive list of all the neuroradiological findings is reported in Table 2.

**Table 2:** Neuroradiological signs in the study population.

Neuroradiological signs	Prevalence (*N*/454)
Optic nerve tortuosity–enlargement	16 (3.5%)
Venous sinuses narrowing/stenosis	15 (3.3%)
Empty sella	13 (2.8%)
Small ventricles	6 (1.3%)
Flattening of the ocular globe	4 (0.8%)
Descended cerebellar tonsils	3 (0.7%)
Optic nerve atrophy	2 (0.4%)
Reduced subarachnoid space	1 (0.2%)
Prominent peri optic subarachnoid spaces	1 (0.2%)
Enlarged cisterna magna	1 (0.2%)
Cerebellar atrophy	1 (0.2%)
General increase in grooves and fissures	1 (0.2%)

### 3.2 Medical treatment

Regarding the management of IIH, medical treatment was the first-line approach except for 15/454 patients (3%) who did not receive any oral medication. In these cases, the reasons were rarely mentioned. For instance, in one patient, medical therapy with acetazolamide was contraindicated due to chronic renal failure in a child undergoing peritoneal dialysis. His condition also contraindicated ventriculoperitoneal shunting, so ultimately he underwent lumbo-pleural shunting with resolution of symptoms.

The first-line medication was acetazolamide in 213/439 patients (48.5%), topiramate in 2/439 (0.5%), and corticosteroids in 13/439 (3%) patients. The co-administration of two drugs simultaneously as the first-line treatment was performed in 17/439 patients (4%): 13/17 patients used ACZ + corticosteroids, 3/17 ACZ + topiramate, and 1/17 ACZ + furosemide.

Second-line medical treatment was administered to 33/439 patients (7.5%): 6/33 topiramate, 2/33 ACZ, 5/33 furosemide, 16/33 steroids, and 1/33 mannitol.

Lastly, before undergoing neurosurgical treatment, 15/439 patients (3.4%) were administered a third drug (12/15 topiramate, 2/15 dexamethasone, 1/15 ACZ + steroid + furosemide). The remaining 194/439 (44%) children were treated with medical therapy, but the specific medication used was not reported, or it was not possible to discriminate between first-, second-, and third-line therapy.

### 3.3 First-line surgical and interventional radiology approach

An invasive approach was performed in 193 out of 454 patients (42.5%), typically following the failure of medical therapy, as reported in Table 3. The most frequently used invasive techniques for treating IIH were shunt placement (lumboperitoneal, LP; ventriculoperitoneal, VP; or ventriculoatrial, VA) (115/193), ONSF (65/193), and VSS (11/193). Less commonly employed techniques included subtemporal or cranial decompression (9/193), endoscopic third ventriculostomy (1/193), and internal cranial expansion (8/193).

**Table 3:** Surgical and interventional radiology procedures in the study population.

Procedures	Single invasive approach (*N*/193)	Multiple approaches (the procedure was a first-line approach, FL)	Total (*N*/193)
Venous sinus stenting	7	4 (1/4 FL)	11
Optic nerve sheath fenestration	60	5 (4/5 FL)	65
Lumboperitoneal shunt	52	11 (6/11 FL)	63
Ventriculoperitoneal shunt	47	7 (2/7 FL)	54
Ventriculoatrial shunt	2	–	2
Lumbopleurical shunt	1	–	1
Internal cranial expansion	4	5 (2/5 FL)	9
Subtemporal decompression	4	4 (3/4 FL)	8

FL, first line.

Of the 193 patients, 177 (92%) underwent only one surgical or interventional radiology procedure. Shunt placement was applied in 115/193 patients (60%) (Table 3), representing the most frequent technique considering LP, VP, and VA together. Among single-shunt procedures, LP was the most frequently used (63/193), and in the majority of patients (52/63), it was the only procedure required to treat IIH. VP shunting was performed in 54/193 children, and it was effective in 47/54 patients. Ventriculoatrial shunting was carried out in only two patients, and both were effective. Lumbo-pleural shunting was performed only in one patient, where medical therapy was contraindicated due to chronic renal failure, as described earlier.

The second most common procedure in our population was ONSF, which was performed in 65/193 patients, particularly in those with visual symptoms. In the majority of patients (60/65, 92%) ONSF was effective, not requiring other interventions, and safe (only one patient experienced a complication, specifically bilateral myelinated retinal nerve fiber syndrome). Twelve of the 65 patients reported the persistence of symptoms (10/12) or the persistence of fundus oculi alterations (2/12), defining the procedure as a failure. Three of these patients (3/12, 25%), however, had previous underlying alterations, such as optic atrophy or congenital cataracts.

VSS was performed in 11/193 patients treated with an invasive approach. Only one patient (0.5%) required a second interventional approach. Ten out of 11 patients who underwent VSS (90%) had documented venous sinus stenosis; for one patient, neuroimaging data on venous sinus stenosis were not available. All but two patients, whose data were not reported, were administered pre- and post-procedural dual antiplatelet therapy and heparinization. Only 1 out of 11 patients required an additional interventional approach.

Cranial subtemporal decompression was performed in 9/193 patients and was the first treatment in 6/9 patients; in the remaining three patients, the procedure was conducted after shunt failure. In 2/9 patients, LP shunt was required as a second invasive procedure.

Eight of the 193 patients underwent internal cranial expansion, a technique that involves removing the skull's inner calvaria table and cancellous bone to increase the volume of the intracranial compartment (14). For 4/8 children, internal cranial expansion was the only neurosurgical approach considered, while in 3/8 it was the second-line approach following the failure of shunting or ONSF. In only one patient, internal cranial expansion required a second-line approach with VSS to resolve symptoms.

### 3.4 Second-line procedures

Sixteen of 193 (8%) children treated with an invasive approach also required a second surgical or interventional radiology treatment.

Among the 63 patients who underwent LP shunting, this was the first-line invasive treatment for 58 patients (92%). The remaining 5/63 patients underwent LP shunting following another procedure's failure: one after VPS, two after subtemporal decompression, two after ONSF, and one after VSS. When LP shunting was performed as a second-line treatment, it was always effective.

Six of 63 (9.5%) patients who underwent LP shunting required second-line surgical treatment. Among these, one patient underwent VP shunting due to LP shunt failure (obstruction or malfunction), while another, with persistent visual symptoms after LP shunting, required ONSF as the second-line approach. Three patients required multiple reinterventions after first-line surgical treatment with LP shunting: in one patient, it was followed by VP shunting and later internal cranial expansion (1/63, 1.5%); in two patients, LP shunting was followed by VP shunting and subtemporal decompression (2/63, 3%). The most severe case of IIH in our study population involved a girl who presented with recurrent headaches. She underwent nine LP shuntings, multiple bilateral subtemporal decompressions, VP shunting with eight revisions, a foramen magnum decompression, and finally an endoscopic third ventriculostomy. Despite these, the patient remained symptomatic and underwent periodic lumbar punctures to control intracranial hypertension (15).

VP shunting showed the highest percentage of reintervention due to obstruction, malfunction or failure, and relapsing of symptoms (13/54, 24%). Six patients (6/54, 11%) only required revision of VP shunting, while the remaining (7/54) required a second invasive approach. Among patients initially treated with VP shunting (49/54, 90%), only two required a second-line procedure. One patient underwent reintervention with VSS and the other with internal cranial expansion.

For the remaining five patients (5/54), VPS was performed as a second-line procedure, and it was effective only in one patient after LP shunting. The other four patients required multiple reinterventions, as reported in Table 3 and Table 4.

**Table 4:** Patients of the study population undergoing at least one second-line invasive treatment.

Author, year	Sex	Age	BMI	Symptoms at onset	Opening pressure (cm H_2_O)	Fundus oculi	Neuroimaging	Medical treatment	Invasive treatment	Outcome
Schwarz et al. (16)	M	7	16.1	Severe headache	32	Mild papilledema	VS intrinsic stenosis	None	I line: ICE II line: VSS	Resolution
	M	16	25.7	Severe headache, tinnitus, diplopia	39	Mild papilledema	VS intrinsic stenosis	ACZ	I line: ONSF II line: VSS	Resolution
Cinciripini et al. (68)	M	5.5	–	Sensory exotropia, severe left eye visual loss	31	Papilledema	Normal	CS and ACZ	I line: ONSF II line: LPS	Resolution
Barrero Ruiz et al. (17)	F	6	–	Severe headache, vomits, impairment of consciousness	55	Normal	Bilateral transverse VS stenosis	ACZ	I line: VSS II line: LPS	After 5 months required LPS revision and removal.
Chang et al. (69)	F	16 (mean)	>25	–	54 (mean)	–	–	ACZ	I line: ONSF II line: LPS	–
Ellis et al. (14)	M	16	22.8	Headache, vertigo	36	Normal	–	–	I line: LPS II line: VPS III line: ICE	No resolution of symptoms
	M	13	22.4	Headache	36	Normal	–	–	I line: VPS II line: ICE	Recurrence of headache
	F	17	45.8	Headache, vision loss	56	Papilledema	–	–	I line: ONSF II line: ICE	Improvement of headache, stable vision, decreased papilledema
Correia et al. (70)	M	7	–	Vomit, irritability, VI nerve palsy	50	Papilledema	Normal	ACZ + furosemide	I line: LPS II line: VPS	Resolution. One revision of VPS.
Beri et al. (71)	F	7	15.5	Headache, visual loss, audiological impairment	27	Normal	Mild stenosis of left transverse VS	I line: ACZ II line: Corticosteroids III line: Topiramate	I line: LPS II line: VPS III line: subtemporal decompression	Postoperative epilepsy
Mandiwanza et al. (15)	F	16	–	Headache	36–40	Papilledema	Chiari I malformation	I line: ACZ II line: Corticosteroids	I line: LPS II line: ICE III line: VPS IV line: foramen magnum decompression V line: cisterna magnum-peritoneal shunt V line: ETV	Recurrence of headache requiring occasional lumbar punctures
Mourani et al. (18)	M	5	–	Headache, VI nerve palsy, visual loss	25	Papilledema	Normal	ACZ and corticosteroids	I line: LPS II line: ONSF	Progression to total blindness. Resolution of headache after kidney transplantation
Hoang et al. (2017) (19)	F	15	36	Headache, diplopia, back pain, urinary retention	>30	Papilledema	–	–	I line: LPS II line: VPS III line: bilateral subtemporal decompression	Resolution after bariatric surgery
Aguilar-Pérez et al. (20)	M	17	–	Headache	50	Papilledema	Stenosis of the right transverse sinus	ACZ	I line: VPS II line: VSS	Resolution
Kessler et al. (72)	F	13	>30	Headache	60	Papilledema	Normal	CS	I line: subtemporal decompression II line: LPS	Resolution
	F	10	>30	Headache, diplopia, VI nerve palsy, visual loss	60	Papilledema	Normal	CS	I line: subtemporal decompression II line: LPS	Resolution

M, male; F, female; ACZ, acetazolamide; CS, corticosteroids; VSS, venous sinus stenting; ONSF, optic nerve sheath fenestration; LPS, lumboperitoneal shunt; VPS, ventriculoperitoneal shunt; VS, venous sinus.

Two children who initially underwent VPS remained symptomatic, and resolution was obtained only with bariatric surgery, which addressed obesity as the main risk factor in these patients (19, 21). Bariatric surgery also proved to be an effective option in a third case, a 15-year-old female who first underwent LP shunting and then required placement of VP shunting and later subtemporal decompression but eventually resolved symptoms only with bariatric surgery (19).

Regarding ONSF, only four patients out of 65 (6%) required an additional treatment: two children received lumboperitoneal shunts, one underwent internal cranial expansion, and one required VSS as a second-line surgical approach. One patient with end-stage renal disease, who was initially treated with LP shunting, underwent ONSF due to relapsing symptoms, but this procedure was unsuccessful. Subsequently, kidney transplantation was needed, and he experienced persistent blindness (18).

As for VSS, 1 of 11 patients (9%) with relapsing symptoms underwent LP shunting. This was a female patient (6 years old.), with severe symptoms (severe headache, decreased level of awareness with hypertonia, and bilateral reactive mydriasis) and an OP of 55 cm H_2_O (17). In three patients (3/11, 27%), VSS was a second-line procedure after ONSF, internal cranial expansion, and VPS, respectively; in all these patients after stent placement, IIH symptoms resolved.

Table 4 summarizes the clinical and radiological characteristics of children who required more than one surgical or interventional radiology approach to control their symptoms.

### 3.5 Focus on venous sinus stenting

VSS was performed in 11 patients and described in five papers (four case series and one retrospective study) (16, 17, 20, 22, 23). As summarized in Table 5, in these patients requiring VSS, MRI showed venous stenosis, particularly in the transverse sinus, superior sagittal sinus, or jugular vein. In only 6/11 patients (55%), it was specified whether the stenosis was extrinsic (smooth gradually narrowing tapered stenosis) or intrinsic (due to arachnoid granulations or fibrous septae). Dual antiplatelet therapy was administered pre-procedural in 9/11 patients, while it was administered in the post-procedural phase in 7/11 patients (Table 5).

**Table 5:** Patients of the study population undergoing venous sinus stenting (VSS).

Author, year	Sex	Age	Symptoms at onset	OP (cm H_2_O)	Fundus oculi	MRI	Prior treatment	Anticoagulant	Trans-stenotic gradient pre- vs. post-stenting (mmHg)	Outcome
Schwarz et al. (16)	F	15	Severe headache, tinnitus, diplopia	65	Papilledema	VS extrinsic stenosis	ACZ	Dual antiplatelet for 1 month; then aspirin for 1 year	34 vs. 1	Resolution
	M	4	Severe headache	32	Mild papilledema	VS intrinsic stenosis	ICE	Dual antiplatelet for 1 month; then aspirin for 1 year	7–7 vs. 1–2 (bilateral stenosis)	Resolution
	F	16	Severe headache	39	Normal	VS intrinsic stenosis	ACZ	Dual antiplatelet for 1 month; then aspirin for 1 year	14 vs. 0	Persistence of mild headache
	F	17	Severe headache	26	Normal	VS extrinsic stenosis	ACZ	Dual antiplatelet for 1 month; then aspirin for 1 year	10 vs. 1	Resolution
	M	16	Severe headache, tinnitus, diplopia	39	Mild papilledema	VS intrinsic stenosis	ONSF	Dual antiplatelet for 1 month; then aspirin for 1 year	19 vs. 1	Resolution
	M	14	Severe headache	33	Normal	VS intrinsic stenosis	ACZ	Dual antiplatelet for 1 month; then aspirin for 1 year	9 vs. 0	Resolution Topiramate after stenting
Carter et al. (22)	F	15	Headache, blurry vision	57	Papilledema	–	ACZ	–	32 vs. 6	Resolution
Dotan et al. (23)	No raw data	No raw data	Headache, no raw data on other symptoms	No raw data	Bilateral severe papilledema	VS stenosis	ACZ ± steroids	–	No raw data	Resolution
Barrero Ruiz et al. (17)	F	6	Severe headache, vomits, impairment of consciousness	55	–	Bilateral transverse VS stenosis	ACZ	Dual antiplatelet therapy	23 vs. 3	Required LP shunting
Aguilar-Pérez et al. (20)	M	17	Headache	50	Papilledema	TS, SSS, jugular vein hypoplasia	ACZ and VPS	Dual antiplatelet therapy pre-stent	14 vs. 0	Resolution
	F	13	Headache	60	Papilledema	Bilateral VS stenosis	ACZ	Dual antiplatelet therapy pre-stent	4 vs./(not available)	Persistent but improving headache

M, male; F, female; ACZ, acetazolamide; VS, venous sinus; ONSF, optic nerve sheath fenestration; VPS, ventriculoperitoneal shunt; TS, transverse sinus; SSS, superior sagittal sinus; ICE, internal cranial expansion.

### 3.6 Outcome

We searched for the outcomes of different procedures but data on their efficacy and effectiveness were lacking. This was due to the types of studies included (mainly case reports and case series) and their retrospective design, the difficulty of comparing data coming from different centers, and the lack of complete information about the outcome. As a matter of fact, for 95 of 193 patients treated with neurosurgical or interventional radiology treatments (49%), outcome information was not available (Table 6).

**Table 6:** Clinical outcome by procedure.

Surgical and interventional radiology procedures	Outcome		
	Positive	Negative	Not specified
Venous sinus stenting	9/11	2/11	–
Optic nerve sheath fenestration	26/65	12/65	27/65
Lumboperitoneal shunt	9/63	9/63	45/63
Ventriculoperitoneal shunt	20/54	14/54	20/54
Ventriculoatrial shunt	–	–	2/2
Lumbopleurical shunt	1/1	–	–
Internal cranial expansion	4/8	4/8	–
Subtemporal decompression	2/9	5/9	2/9

Positive outcome, resolution or improvement of symptoms; negative outcome, persistence or worsening of symptoms.

In children who underwent more than one invasive approach, we counted failed procedures requiring reintervention among negative outcomes. Sevnty-one of 193 (37%) children had a positive outcome, showing complete resolution of symptoms, normalization of visual acuity and papilledema (46/71, 65%), or at least improvement of symptoms (25/71, 35%). Considering single procedures, we observed a positive outcome in 9/11 (82%) children treated with VSS, 26/65 (40%) ONSF, 9/63 (14%) LPS, and 20/54 (37%) VPS.

We considered patients with a negative outcome as those with persistence or worsening of symptoms, pathological fundus oculi, or altered visual field examination (i.e., showing optic atrophy, pale optic disc, enlarged blind spots). Only one patient (1/194, 0.5%) showed bilateral myelinated retinal nerve fibers syndrome as a collateral effect of ONSF. Overall 27/193 patients showed a negative outcome (14%). Examining the individual procedures, a negative outcome was reported in 2/11 (18%) children who underwent VSS, 12/65 (18%) ONSF, 9/63 (14%) LP shunting, and 14/54 (26%) VP shunting. None of the patients died during follow-up for IIH, and the worst outcome was blindness (in one patient) (18).

## 4 Discussion

### 4.1 Clinical presentation

Pediatric IIH is a rare and severe condition with an overall incidence of about 1 case per 100,000 children in the general population. The incidence increases during the second decade of life, particularly between 12 and 15 years of age (24, 25). In our review, we found a female sex prevalence (66%) and a median age at onset of 12 years.

The data of our pediatric population were analyzed according to the original Dandy criteria (6), considering an opening pressure of >25 cm H20 (rather than >28 cm H_2_O). This cutoff remains currently used for diagnosing IIH in both adults and children without sedation during lumbar puncture and without obesity. The papers included in this review never specified whether patients were sedated and only occasionally mentioned obesity, and this lack of information could represent a potential bias. Risk factors such as obesity, medical treatments (i.e., growth hormone, corticosteroid withdrawal, lithium, all-trans retinoic acid, or isotretinoin), and systemic conditions (i.e., endocrinological and celiac disease) are quite common in patients presenting with IIH symptoms (26). In most of our study population, symptom resolution was achieved after the removal of risk factors (i.e., discontinuing growth hormone administration or treating obesity). The most frequent symptoms at onset included headache with or without nausea and vomiting, visual symptoms (such as diplopia, blurry vision, and transient visual obscuration), neck/shoulder pain, and tinnitus, while the most common clinical signs included reduced visual acuity or visual field and VI nerve palsy, as previously reported (9).

Unilateral or bilateral papilledema was found in the majority of our patients (90%), but it is important not to exclude IIH diagnosis in the case of normal fundus oculi and to suspect it based on clinical presentation. In previous literature, papilledema was detected even less frequently, ranging from 66% to 82.2% (27, 28). Although optic nerve swelling is a nonspecific finding and may be absent (e.g., in children with optic atrophy), the diagnostic role of this examination is well acknowledged (24, 29). Some studies in adults have documented a relationship between the grade of optic disc edema and intracranial pressure values (30–32), while these data are not available for pediatric series nor in our study population.

### 4.2 Neuroimaging and other diagnostic techniques

**Figure 2:**
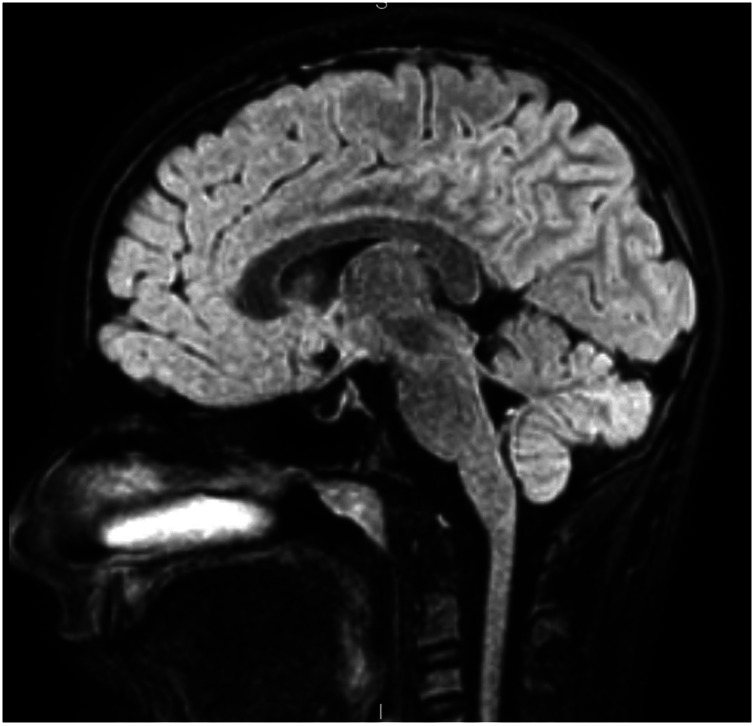
A 12-year-old male patient with partial empty sella aspect in the sagittal sequence.

**Figure 3:**
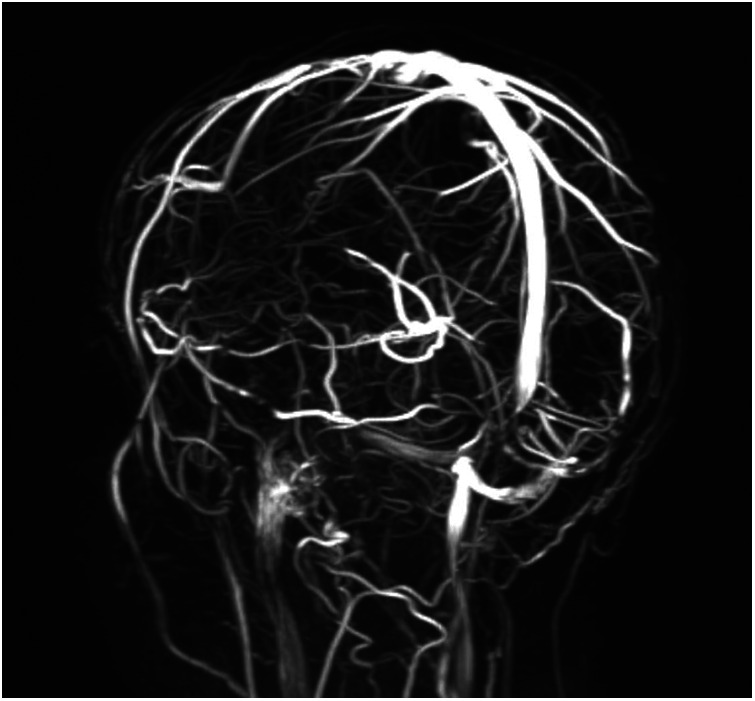
An 11-year-old female patient with reduced flow signal in the transverse sinuses and at the passage between transverse and sigmoid sinuses at the MR venography.

**Figure 4:**
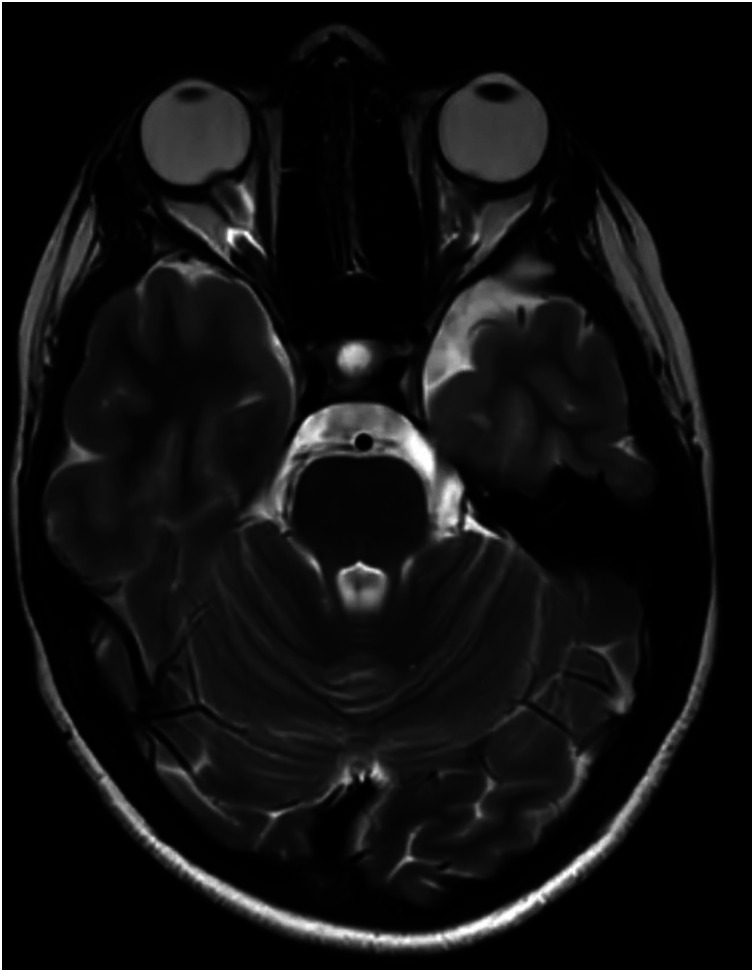
An 11-year-old female patient with optic nerve tortuosity and bilateral protrusion of the papillae in the axial T2w sequence.

Revised diagnostic criteria for IIH diagnosis require the presence of five criteria: papilledema, raised intracranial pressure (>25 cm H_2_O in unsedated and not obese children, or >28 cm H_2_O if sedated or obese), a normal neurological examination except for cranial nerve abnormalities, normal CSF composition, and the exclusion of secondary causes with neuroimaging (8). Neuroimaging is mandatory to exclude secondary causes of increased intracranial pressure. Furthermore, the progressive improvement of MRI has permitted over time the identification of neuroradiological signs that are suggestive of IIH (33). If the aforementioned criteria are not fulfilled (i.e., absence of papilledema or sixth nerve palsy), a definite diagnosis is also possible if at least three of the following neuroradiological signs are present: empty sella (Figure 2), flattening of the posterior aspect of the globe, transverse sinus stenosis (Figure 3), and distension of the perioptic subarachnoid space with or without a tortuous optic nerve (Figure 4). The presence of more than three of the abovementioned neuroimaging features has been correlated to the severity of visual impairment. According to the literature, optic nerve tortuosity was the most sensitive sign of IIH, while the flattening of the posterior globe and distension of the perioptic subarachnoid space displayed the highest specificity (34). Our results confirm that the most frequent neuroradiological findings are optic nerve tortuosity or enlargement and empty sella but also venous sinus narrowing or stenosis.

Some of these MRI abnormalities may be partially attributed to the effects of general anesthesia (35–37); therefore, the presence of three or more features is less likely to be influenced by sedation and can be considered more specific for IIH (35), increasing the likelihood of an IIH diagnosis (38).

Recently, cerebral angiography and venography have also demonstrated an important diagnostic role in measuring intracranial venous pressure prior to VSS, confirming the presence of a pressure gap between the jugular vein and transverse or sagittal venous sinuses. This evaluation is typically performed after non-invasive imaging to rule out other etiologies of increased ICP. Based on this examination, they are eligible for VSS (39, 40). While no specific pressure gradient threshold has been established in the literature, most studies report using a pressure gradient of >8 mmHg as a criterion to consider venous sinus stenting in the adult population (40). No data are available for pressure gradient and eligibility for VSS in IIH in children. In our experience, the mean trans-stenotic gradient pre-VSS is 15.18 mmHg (range, 4–34), and only 3/11 patients showed a gradient of <8 mmHg.

Finally, as in many other diagnostic processes of pediatric pathologies, ultrasound has gained widespread approval also in the diagnosis of IIH thanks to its safety, repeatability, and lack of radiation exposure (41). Ultrasound finds application in this setting through measurement of optic nerve sheath diameter (ONSD) and transcranial Doppler. Both methods are useful in the diagnostic setting, showing how the variation of ONSD and baseline blood flow in brain blood vessels correlate significantly with an increase of intracranial pressure (42, 43), but might also be valid non-invasive methods for longitudinal monitoring in the follow-up of pediatric patients with a previous diagnosis of IIH (44, 45).

### 4.3 Medical treatment

For patients with IIH and visual symptoms, acetazolamide is used as first-line treatment in the majority of patients (25, 46). This approach was also commonly applied in our population: 52.4% of patients were treated with acetazolamide either as a single (48.5%) or combined therapy (3.9%). Acetazolamide can improve symptoms and visual outcomes by reducing the rate of CSF production [by 6% to 50% (9)].

Acetazolamide is considered the best drug to treat IIH symptoms. More specifically, the IIH randomized controlled treatment trial conducted by the NORDIC consortium (9, 47) on adult patients demonstrated that acetazolamide was effective for mild visual loss with improvements in perimetric mean deviation, papilledema severity, CSF opening pressure, weight loss, and quality of life. Ball et al. (48) documented a positive effect of the drug on headaches and visual disturbances such as transient visual obscuration and binocular contrast sensitivity. However, no significant differences were found in papilledema, headache severity, visual acuity, and visual field. In conclusion, both trials showed a lack of evidence for effectiveness in improving visual acuity and reducing headache severity (9, 48, 49).

Patients who do not respond or cannot tolerate acetazolamide due to its multiple side effects (metallic taste, tingling sensation in the mouth, paresthesia, and nausea) may require other drugs (10, 50). In our study population, topiramate, furosemide, and corticosteroids have also been administered (much less frequently than acetazolamide), either as monotherapy or as combination therapy. However, none of these treatments have been evaluated in randomized controlled trials and the majority of the available data comes from case reports or series. Topiramate has weak carbonic anhydrase inhibition properties, making its mechanism of action similar to that of acetazolamide; it may work better as an “add-on” to acetazolamide (50).

Furosemide has weak carbonic anhydrase inhibiting properties, but its main mechanism is probably due to its diuretic effect combined with a reduction of sodium transport into the brain (51).

Corticosteroids are used in cases of worsening or persistent visual symptoms or refractory severe headaches despite treatment with acetazolamide. They should be administered for short courses of no more than a few weeks. Corticosteroids are thought to reduce vasogenic edema and intracranial pressure but their efficacy remains unproven (52).

### 4.4 Surgical and interventional radiology procedures

An invasive approach should be pursued in cases of refractory IIH despite a maximum medical therapy, particularly in cases of significant visual loss at onset and declining visual function (47, 53). Currently, the main surgical options described in the literature for IIH treatment include ONSF, VSS, and CSF diversion (mainly lumboperitoneal and ventriculoperitoneal shunt). Since there is no evidence favoring any specific treatment, both in adult and pediatric populations, the choice of technique mostly depends on local availability and expertise (54).

CSF diversion with shunt placement (LP, VP, ventriculoatrial, or lumbo-pleural) is one of the most frequently applied approaches in both children and adults with medically refractory IIH cases, providing immediate improvement in both headaches and visual involvement (24, 25, 55).

This trend is reflected in our study as well: CSF diversion was the most commonly employed technique (115 patients for a total of 120 procedures), and, considering the specific procedure, LP shunt (63/120, 53%) slightly prevailed over VP shunt (54/120, 45%). These procedures carried a notable risk of complications: in pediatric patients, shunt failure is experienced in 30%–40% of cases within 1 year and approximately 50% within 2 years (56). In our review, the need for revision or retreatment was much lower (20/115, 17%) and concerned 8/63 (11%) LP shunt and 12/54 (23%) VP shunt-treated patients. The reasons for revision or retreatment reported in our cases align with those reported in the literature (shunt failure, dislocation, obstruction, infection, and overdrainage).

ONSF is considered in several papers as the first-line procedure when vision involvement is the primary concern (4, 47). This technique provides drainage of cerebrospinal fluid from the subarachnoid space around the optic nerves (57), reducing pressure on the optic disc at the level of the lamina cribrosa. Moreover, this technique has the advantage of leading to a bilateral visual recovery even when only one eye is operated on; in fact, the visual outcome frequently improves in the non-operated eye too, avoiding the necessity for a second surgery and all the associated risks (58). In our series, it was the second most applied approach with 65 described cases (first, if considering each CSF diversion procedure separately). In our study, this technique has fewer complications than CSF diversion [only one case of acquired bilateral myelinated retinal nerve fibers has been described (59)]. 6/65 (9%) patients experienced persistence of visual symptoms after ONSF, likely because the intervention was performed when visual deterioration had already progressed to a severe stage and was associated with permanent damage. Moreover, 3/6 patients had underlying optic alterations (optic nerve atrophy, congenital cataracts) that likely influenced the outcome. Finally, the assessment and monitoring of optic nerve dysfunction in children are challenging, which may contribute to delays in intervention.

VSS is the most recently developed technique, and it has shown promising results in improving both visual and headache outcomes (60). Its role in IIH treatment is supported by the presence of areas of focal stenosis within the dural venous sinus system in 68%–93% of patients, as described in recent studies (22). The etiology of this stenosis is still a matter of open discussion: some studies suggest the presence of undiagnosed structural lesions of the venous sinuses (61), and others propose a possible role of non-occlusive microthrombosis lining dural vessels that might impede CSF absorption (62). This may lead to cerebral venous hypertension, which could be the cause rather than a consequence of a raised CSF pressure (63). In our study, stenosis of the venous sinus system was documented by MRI in almost all the studied patients (10 out of 11 total VSS cases). The stenosis has been implicated as either a primary or contributory cause of IIH and targeted treatment of the stenotic sinus through stent placement or venoplasty alone has emerged as a new promising treatment strategy (64). In our review, among the 11 patients treated with VSS, 9/11 (82%) showed good efficacy resulting in complete resolution or remarkable improvement in their onset symptoms, and only one patient out of 11 required a second-line invasive approach. As highlighted by recent meta-analyses conducted in the adult population (65, 66), the VSS approach is associated with a lower revision rate compared to other procedures (such as CSF diversion procedures), thus reducing the potential morbidity and mortality of IIH. In our study population, no procedural complications or revisions were described, suggesting that VSS is also safe in the pediatric population. However, the studies included in our review are mostly based on case reports or small series of VSS placement recently performed [except for that of Aguilar et al. (20), which is the most dated]. Therefore, data on long-term patency in pediatric patients remain limited.

### 4.5 Outcome

In our study population, with comparison limits, VSS appears to yield the most favorable results in terms of headache resolution and visual outcome (9/11, 82%), followed by ONSF (26/65, 40%), VPS shunting (20/54, 37%), and LPS shunting (9/63, 14%). Our results align with those presented by A. Kalyvas et al. in their most recent systematic review of the surgical approach to IIH treatment in the adult population (60). Based on a substantial number of observational studies, the authors concluded that VSS provided the best results in terms of symptom resolution and visual outcome, and ONSF appeared to be efficient in preventing further visual deterioration and improving visual outcome while CSF shunting, despite being the most commonly performed procedure, associated with high failure and complication rates.

The most concerning outcome in pediatric IIH is irreversible visual impairment, with permanent visual loss or visual field deficit that may affect up to 20% of children (67). In our study, persistent visual dysfunction despite medical and/or surgical therapy was observed in a minority of cases [19/454, (4%)], all of whom presented with visual symptoms at onset. Complete blindness at follow-up was the worst visual outcome and it occurred in one patient (1/194%–0.5%) (18); none of the patients died during follow-up. These data highlight once again the importance of enhanced awareness, early diagnosis, and prompt treatment to avoid a negative outcome and, particularly, permanent visual damage.

## 5 Study limitations

We conducted a literature review over a wide time range (the last 30 years) and observed that the available papers only refer to case series and single case reports. These papers refer to different centers, where patients were treated with variable invasive approaches based on individual center experience and on the clinical presentation. This represents the main limitation of the study. However, the intrinsic characteristic of the pathology, with severe and acute presentation, makes it difficult to conduct multicentre prospective randomized controlled studies.

Another limitation of our study is the small sample size of patients included and the incomplete data in some studies, where detailed information was not available. In some papers (i.e., wide case series), we also had to rely on pooled data or average values. Aware of this limitation and to be as clear as possible, we always specified when data was not complete in our results section. Moreover, when available, we always extracted as much data as possible from the tables and supplementary materials, thus maintaining the paper in the selection.

In conclusion, to focus on neurosurgical and interventional approaches to IIH, we selected articles including data about pediatric patients surgically treated. These works concern the most severe cases or the most difficult to treat with medical therapy alone and do not reflect the general population of patients with IIH. As a result, in our review, it seems that there is an elevated percentage of invasive intervention compared to real life, whereas the medical approach is considered the first-line treatment and is generally effective.

## 6 Conclusion

IIH is typically a benign condition that can generally be managed and resolved with medical therapy alone if an early diagnosis is correctly made. Nevertheless, not infrequently and particularly in the most severe cases (characterized by significant neurological and visual deterioration at onset), invasive approaches such as neurosurgery and/or interventional radiology are needed. In pediatric cases, similarly to adults, the most commonly used techniques include CSF shunting, ONSF, and VSS.

CSF shunting is the longest-established technique and remains the most frequently applied as it is easily accessible in most centers.

ONSF has demonstrated good results in terms of safety also in the pediatric population. According to our literature review, this could be the preferred approach when a rapid decline in visual function occurs and the probability of a full recovery depends on the promptness of the intervention.

VSS is the most recently introduced approach in the management of pediatric IIH and is primarily indicated in cases with stenosis of the intracranial venous system. In our review, VSS showed good results in terms of symptom resolution and the need for reintervention, although the number of cases remains limited and its application is still restricted to a few centers.

The VSS technique appears to have a promising outcome, but much remains to be studied regarding its superiority over the established approaches for which international experience is more solidly grounded.

Further studies are needed to better understand the physiopathology underlying IIH to optimize therapeutic strategies for pediatric IIH and to individualize treatment, with a rational approach based on patient stratification.

## Data Availability

The original contributions presented in the study are included in the article/Supplementary Material, further inquiries can be directed to the corresponding author/s.
